# Decreased macular choriocapillaris in thyroid-associated ophthalmopathy: focusing on chorioretinal folds with and without optic disc edema

**DOI:** 10.3389/fendo.2023.1123820

**Published:** 2023-04-21

**Authors:** Peng Zeng, Jia-qi Liang, Yuan-yu Peng, Shu-xian Fan, Jing Wang, Shi-you Zhou, Peng Tian, Mei Wang

**Affiliations:** ^1^ Department of Ophthalmology, Sun Yat-sen Memorial Hospital, Sun Yat-sen University, Guangzhou, China; ^2^ State Key Laboratory of Ophthalmology, Zhongshan Ophthalmic Center, Sun Yat-sen University, Guangzhou, China; ^3^ Department of Otolaryngology, Sun Yat-sen Memorial Hospital, Sun Yat-sen University, Guangzhou, China

**Keywords:** thyroid-associated ophthalmopathy, chorioretinal folds, optic disc edema, choriocapillaris, vessel density

## Abstract

**Purpose:**

The aim of this study was to evaluate the vessel density (VD) of the macular choriocapillaris (CC) and retina in thyroid-associated ophthalmopathy (TAO) patients with chorioretinal folds (CRFs) with and without optic disc edema (ODE) and the correlations of these characteristics with visual function.

**Method:**

This was a cross-sectional study. Twenty TAO patients with CRFs (35 eyes) and 20 normal subjects (normal group, 40 eyes) were recruited at the Ophthalmology Department of the Sun Yat-sen Memorial Hospital from March 2018 to October 2022. Then, CRF patients were divided into two groups, the ODE and non-ODE groups (NODE), based on the presence or absence of ODE. All the patients underwent optical coherence tomography angiography (OCTA) and the VD of the macular CC and retina was computed. The correlation of VD and visual function was analyzed.

**Results:**

Compared with the normal group, the macular whole-image VD in the retinal superficial layer (SLR-mwiVD: 49.82 ± 3.38 in the normal group, 42.44 ± 5.40 in the NODE group, and 42.51 ± 5.37 in the ODE group), deep layer (DLR-mwiVD: 51.05 ± 6.23 in the normal group, 45.71 ± 6.66 in the NODE group, and 46.31 ± 5.48 in the ODE group), and CC (CC-mwiVD: 70.23 ± 2.47 in the normal group, 68.04 ± 3.73 in the NODE group, and 63.09 ± 6.51 in the ODE group) was decreased in the NODE (all *p* < 0.05) and ODE group (all *p* < 0.01). There was no difference in these parameters except CC-mwiVD between the ODE and NODE groups. The CC-mwiVD in the ODE group (63.09 ± 6.51) was significantly reduced compared with that in the NODE group (68.04 ± 3.73, *p* = 0.004). All these VD parameters were negatively correlated with BCVA, VF-PSD, and P100 latency and positively associated with VF-MD, P100 amplitude, and HRR scores (all *p* < 0.05).

**Conclusions:**

There was a significant decrease in the VD of the macular CC and retina of patients with CRFs with or without ODE, which was correlated with visual dysfunction. The VD of the macular CC in CRF patients with ODE was significantly reduced compared with that in the NODE group, but similar results were not observed in the retina.

## Introduction

Thyroid-associated ophthalmopathy (TAO) is an autoimmune disorder related to thyroid dysfunction that can threaten the vision in severe stages. Chorioretinal folds (CRFs), corrugations at the level of the choroid, Bruch’s membrane, retinal pigment epithelium, and the overlying neurosensory retina, have been described in a wide range of ophthalmic and systemic diseases, including age-related macular degeneration (1), posterior scleritis (2), orbital tumors (3), hypotony (4), hyperopia (5), central serous chorioretinopathy (6), and TAO (7). Previous studies have demonstrated that CRFs are often an indicator of conditions that threaten vision in patients with TAO (7–11). The mechanism underlying CRF formation in TAO is not clearly understood but is hypothesized to be related to orbital hypertension, which may place compressive stress on the choroid, Bruch’s membrane, and optic nerve; the latter results may be related to retinal and choroidal microvascular changes (1).

Optical coherence tomography angiography (OCTA), a noninvasive and quantitative technique, has been used to evaluate retinal and choroidal microvascular density (VD) in TAO patients (12–17) and has been used to diagnose and monitor the early stage of vision-threatening TAO (18–21). Our previous study on OCTA reported that the vessel density (VD) of the retinal macula and radial peripapillary capillaries (RPCs) was decreased in CRF patients compared with TAO patients without CRFs (11). However, these CRF patients were unselected in a previous study, including CRF patients with or without optic disc edema (ODE). As is known, ODE is a specific sign to diagnose dysthyroid optic neuropathy (DON) (22–24). A recent study has shown a reduction in VD of choroidal RPC in DON eyes, and this is correlated with visual field (VF) defects (18). Therefore, CRF patients with ODE should be diagnosed as DON and there is a logical reason to suggest that CRF patients with or without ODE may influence the retinal and choroidal VD changes in TAO patients. However, as far as we know, no report has described changes in the macular choroidal VD in TAO patients with CRFs with or without ODE. Therefore, OCTA was implemented to detect the changes in the VD of macular retina and choriocapillaris (CC) in TAO patients with CRFs with and without ODE and to analyze the relationship between these changes and visual function parameters in this study.

### Patients and methods

The cross-sectional study was conducted at Sun Yat-sen Memorial Hospital, Sun Yat-sen University, from March 2018 to October 2022. This study was conducted in accordance with the tenets of the Declaration of Helsinki, and the protocol of this study was approved by the Sun Yat-sen University Sun Yat-sen Memorial Hospital Ethical Committee in the People’s Republic of China.

Twenty TAO patients with CRFs were diagnosed in the Department of Endocrinology and Ophthalmology at Sun Yat-sen Memorial Hospital, Sun Yat-sen University based on the European Group on Graves’ Orbitopathy (EUGOGO) criteria (22, 25). Twenty normal subjects (40 eyes) were enrolled as controls in this study. The inclusion criteria of CRF patients were as follows: (1) age >18 years; (2) refractive errors with spherical equivalent (SE) < −6 diopters; (3) clear refracting media; and (4) no history of treatment with large-dose systemic glucocorticoids, immunosuppressive agents, or ocular radiation therapy. The exclusion criteria were as follows: (1) any other systemic diseases except TAO, such as diabetes and systemic hypertension; and (2) any history of ocular diseases, prior ophthalmic surgery, or trauma.

The clinical data included age; sex; thyroid function, including free triiodothyronine (FT3), free thyroxine (FT4), thyroid-stimulating hormone (TSH), and thyrotrophin receptor antibody (TRab); signs, symptoms, and duration of TAO; history and duration of thyroid disease; and the presence of other systemic and eye diseases. Ophthalmic examinations included best-corrected visual acuity (BCVA) measured with a standardized logMAR visual acuity chart, slit lamp examination of the anterior segment, and fundus examination. Proptosis was measured with a Hertel exophthalmometer (Oculus, Germany), and intraocular pressure (IOP) measurements (Canon TX-20, YZB/JAP3501-2012, Tokyo, Japan) were obtained in the primary position. Standard automated VF and pattern visual evoked potential tests were performed using the Humphrey automated visual field analyzer (Program 30-2, Humphrey Field Analyzer II 750; Carl Zeiss Meditec, Inc., Dublin, CA, USA) and a pattern visual evoked potential analyzer (ESPION; Diagnosys LLC, Inc., Cambridge, UK), respectively. Color vision scores were tested by the Hardy-Rand-Rittler color plates (HRR, Richmond products, Inc. Albuquerque, NM, USA). Blood pressure and heart rate were measured after participants rested for at least 5 min. Mean arterial blood pressure (MABP) was computed as diastolic blood pressure (DBP) plus one-third of the patient’s pulse pressure.

**Figure 1:**
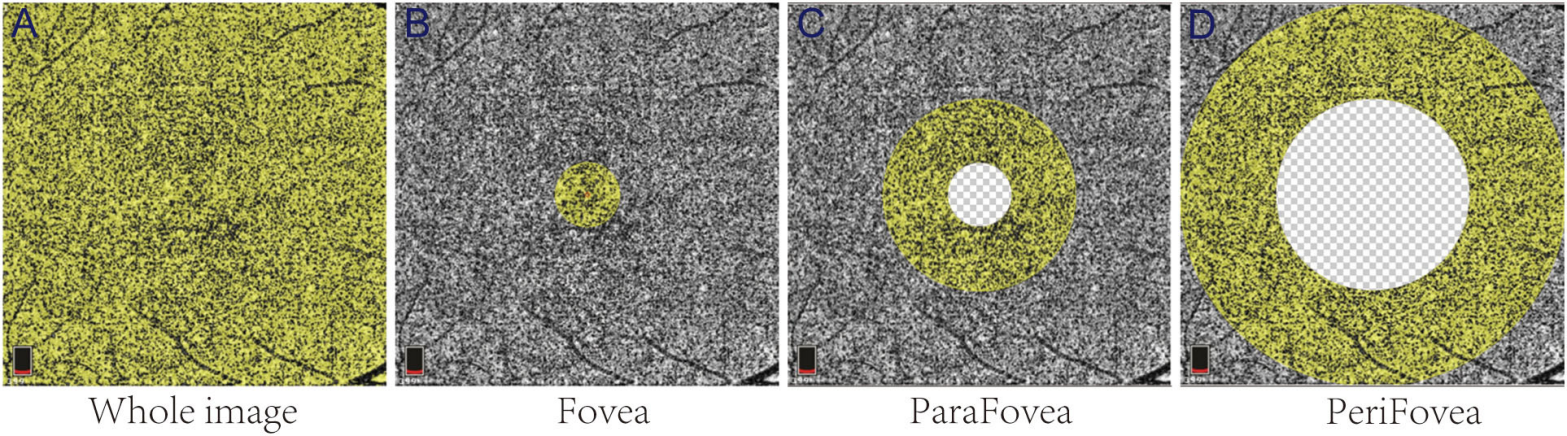
Representative partition images of the macular choriocapillaris. The yellow areas in **(A–D)** represent the whole-image, Fovea, ParaFovea, and PeriFovea regions, respectively.

OCTA images were obtained using prototype AngioVue software 2.0 of the RTVue XR Avanti spectral domain OCT device (Optovue, Inc., Fremont, CA, USA). The VD parameters of the macular retinal superficial layer (SLR), deep layer (DLR), and CC were obtained from the 6 mm × 6 mm area of scan centered on the fovea. The area of macular scanning was segmented by an annular grid into three fields: the foveal, parafoveal, and perifoveal zones. Representative partition images in the CC are shown in **Figure 1**. In addition, the nerve fiber thickness of the macula and optic disc were assessed using ganglion cell complex (GCC) mode and optic nerve head mode, respectively. All OCTA imaging was performed by a trained ophthalmic examiner. Low-quality OCTA images (SSI < 60 or scan quality < 6) were excluded.

### Statistical analysis

Statistical analysis was performed using SPSS (Statistical Package for Social Sciences; SPSS Inc. IBM, Armonk, NY) version 26.0. The mean ± standard deviation was used for continuous variables and frequency (%) was used for classification variables. Data with a normal distribution were evaluated by independent sample *t*-tests, data with nonnormal distribution were evaluated by Mann–Whitney test, and categorical variables were evaluated by chi-square analysis. Clinical data were compared and each eye was included in the study as an independent sample. Considering the correlation between the eyes of individual patients, a generalized estimation equation (GEE) was used to increase the statistical power. Pearson correlation analysis was used to analyze the correlation between visual function and VD parameters. Differences were considered statistically significant at *p* ≤ 0.05.

## Results

### Demographic and clinical data

A total of 20 normal subjects (normal group) and 20 CRF patients (CRF group) were enrolled in the study, and their clinical data are listed in **Table 1**. More than two-thirds in CRF patients were male (17 patients, 85%), smoked (14 patients, 70%), and had onset in both eyes (15 patients, 75%). The most common primary thyroid disease of CRF patients was hyperthyroidism. The durations of autoimmune thyroid diseases and TAO were 12.65 ± 7.82 and 9.40 ± 4.68 months, respectively. CRF patients had significantly higher TSH and TRab level than the normal group (all *p* < 0.05). There was no significant difference in age, sex, systolic blood pressure (SBP), DBP, MABP, heart rate, FT3, or FT4 between the two groups.

**Table 1:** Demographic and clinical data.

Variables	Normal	CRFs	*p*
*N*	20	20	NA
**Sex**, male, *n* (cases, %)	17, 85%	17, 85%	NA
**Age** (years, mean ± SD)	46.40 ± 10.54	47.95 ± 12.00	0.667
**SBP**, mmHg	124.05 ± 11.22	123.00 ± 6.10	0.715
**DBP**, mmHg	78.60 ± 7.54	79.10 ± 5.21	0.808
**MABP**, mmHg	93.75 ± 8.19	93.73 ± 4.73	0.994
**Heart rate**, times/min	83.20 ± 10.12	82.95 ± 9.08	0.935
**Smoking status** (smoker, %)	0, 0%	14, 70%	NA
**Primary thyroid disease** (hyperthyroidism, %)	–	20,100%	–
**Duration of autoimmune thyroid disease**, months	–	12.65 ± 7.82	–
**TAO duration**, months	–	9.40 ± 4.68	–
**Thyroid** function **test at presentation**			
** FT3, pmol/L**	5.16 ± 0.51	6.81 ± 6.20	0.243
** FT4, pmol/L**	15.56 ± 2.28	22.72 ± 24.43	0.200
** TSH, mU/L**	1.85 ± 1.17	1.33 ± 1.84	0.030
** TRab, U/L**	0.88 ± 0.23	14.13 ± 11.65	<0.001

Data are shown as the mean ± SD. N, number of patients; SBP, systolic blood pressure; DBP, diastolic blood pressure; MABP, mean arterial blood pressure; FT3, free triiodothyronine; FT4, free thyroxine; TSH, thyroid-stimulating hormone; TRab, thyrotrophin receptor antibody; NA, not applicable.

### Comparisons of ophthalmic parameters

Twenty normal subjects (40 eyes) and 20 CRF patients (35 eyes) were divided into the normal group (40 eyes), CRFs with ODE group (ODE group, 18 eyes), and CRFs without ODE group (NODE group, 17 eyes), and the groups are described in **Table 2**. Regardless of ODE status, CRF patients had significant differences in BCVA, IOP, proptosis, VF-MD, P100 latency, P100 amplitude, and HRR scores compared with control group (all *p* < 0.05), and these parameters were not different between the ODE and NODE groups (all *p* > 0.05). The VF-PSD in the ODE group was significantly higher than that in the NODE (*p* = 0.037) and normal groups (*p* < 0.001). The thickness of the RNFL and GCCL in the ODE group was obviously thicker than that in the NODE and normal groups (all *p* < 0.05), and these parameters were not different between the ONDE and normal groups. There was no difference in CAS scores between the ODE and NODE groups.

**Table 2:** The comparisons of ophthalmic parameters.

Variables		CRFs		Post-Hoc Analysis *p*-Values			
	Normal *n* = 40 eyes	NODE *n* = 17 eyes	ODE *n* = 18 eyes	*p*	Normal vs. NODE	Normal vs. ODE	NODE vs. ODE
**BCVA**, logMAR	−0.03 ± 0.06	0.20 ± 0.34	0.29 ± 0.28	<0.001	0.004	<0.001	0.384
**SE**, diopter	0.03 ± 0.56	0.29 ± 1.50	0.14 ± 1.48	0.729	0.458	0.742	0.745
**IOP**, mmHg	14.87 ± 2.21	23.35 ± 7.56	20.78 ± 7.04	<0.001	<0.001	<0.001	0.283
**Proptosis**, mm	15.78 ± 1.82	21.64 ± 2.26	20.78 ± 4.40	<0.001	<0.001	<0.001	0.438
**VF-MD**, Db	−0.16 ± 0.63	−6.20 ± 6.52	−8.65 ± 5.68	<0.001	<0.001	<0.001	0.211
**VF-PSD**, Db	1.53 ± 0.30	3.46 ± 2.27	5.14 ± 2.77	<0.001	<0.001	<0.001	0.037
**P100** latency, ms	100.96 ± 4.27	111.24 ± 7.49	118.86 ± 9.67	<0.001	<0.001	<0.001	0.838
**P100** amplitude, lV	13.62 ± 4.68	5.52 ± 2.73	5.33 ± 1.33	<0.001	<0.001	<0.001	0.799
**HRR scores**	19.50 ± 0.51	9.64 ± 9.12	11.47 ± 8.31	<0.001	<0.001	<0.001	0.529
**RNFL,** μm	113.62 ± 13.79	112.35 ± 15.86	180.94 ± 76.13	<0.001	0.791	<0.001	<0.001
**GCCL,** μm	95.99 ± 12.99	95.65 ± 8.63	104.67 ± 15.44	0.068	0.900	0.033	0.028
**CAS**	–	2.47 ± 1.77	3.11 ± 1.97	0.320	–	–	–

Data are shown as the mean ± SD.

### Comparisons of the VD in macular retina and choriocapillaris

**Figure 2:**
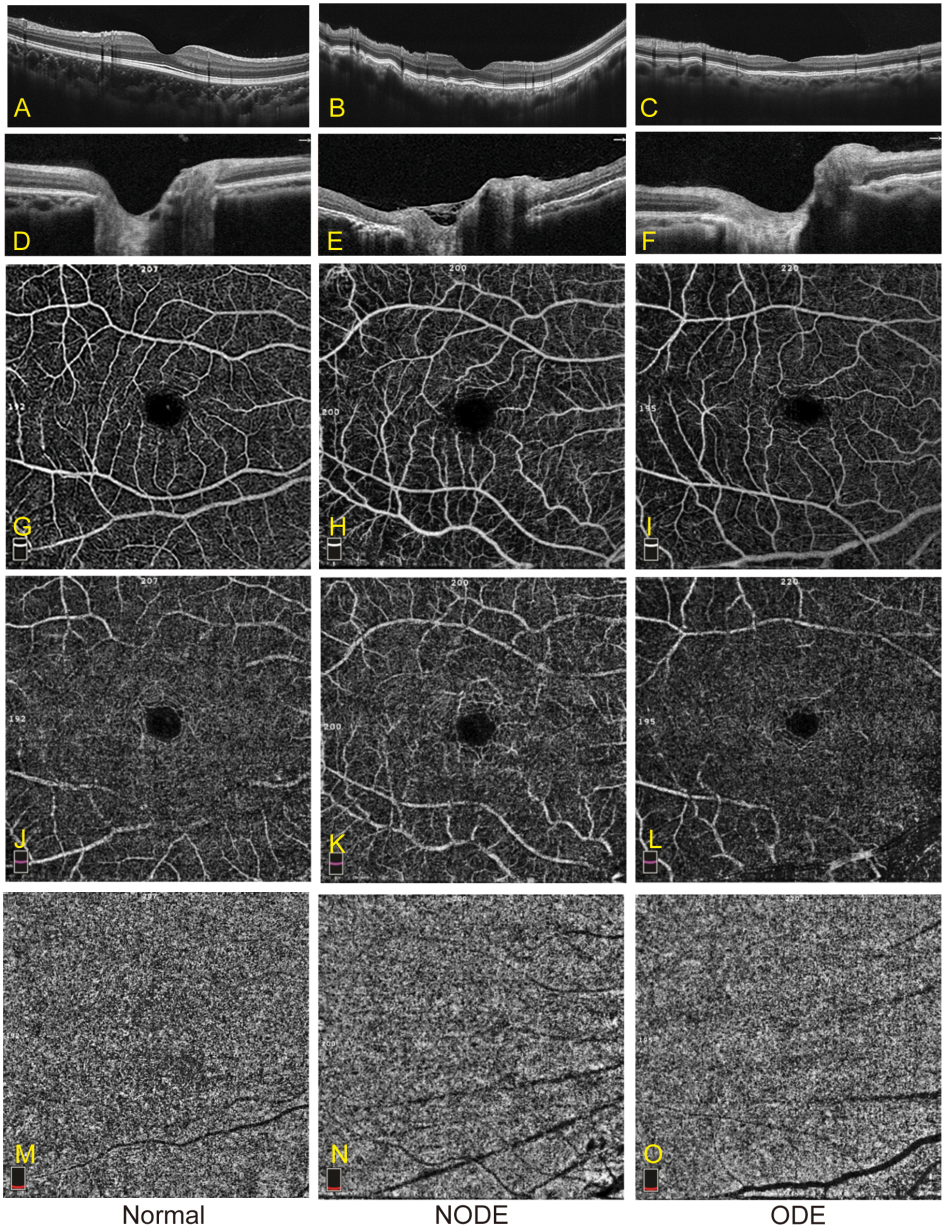
Representative images of OCTA in the normal, NODE, and ODE groups. **(A–C)** show optical coherence tomography (OCT) images of the macula in the normal, NODE, and ODE groups, respectively. The optical coherence tomography (OCT) images of the optic nerve head in the normal, NODE, and ODE groups are displayed in **(D–F)**, respectively. Macular SLR vessel density map is displayed with the normal **(G)**, NODE **(H)**, and ODE **(I)** groups. **(J–L)** exhibit macular DLR vessel density map in the normal, NODE, and ODE groups, respectively. **(M–O)** show macular CC vessel density in the normal, NODE, and ODE groups, respectively. SLR, superficial layer; DLR, deep layer; CC, choriocapillaris.

**Figure 3:**
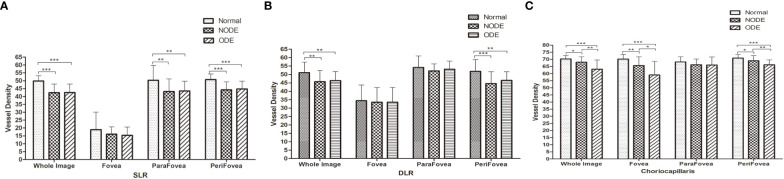
Macular VD of SLR, DLR and CC in different groups were listed in **(A)**, **(B)** and **(C)** respectively. Compared with normal group, there is a significant decreased in VD of macular CC and retina in NODE and ODE group. The VD of macular CC in ODE group was significantly reduced than that of NODE group, while not in macular retina. SLR, superficial layer; DLR, deep layer; CC, choriocapillaris. (* indicates p<0.05, ** indicates p<0.01 and ***indicates p<0.001).

Representative OCTA images of the three groups are shown in **Figure 2**. The VD parameters of the macular retina and CC in the normal, NODE, and ODE groups are shown in **Table 3** and **Figure 3**. In the SLR, the VD of the whole image (both *p* < 0.001), ParaFovea (both *p* < 0.01), and PeriFovea (both *p* < 0.001) but not the Fovea (both *p* > 0.05) was significantly decreased in the ODE and NODE groups compared with the normal group. There was no difference in these parameters between the ODE and NODE groups (all *p* > 0.05). In the DLR, the whole image and PeriFovea VD were significantly decreased in the ODE (both *p* < 0.01) and NODE groups (both *p* < 0.01), compared with the normal group. There was no difference in the VD of the Fovea and ParaFovea among three groups (all *p* > 0.05). In the CC, compared to the normal group, the VD of the whole image (both *p* < 0.05), Fovea (both *p* < 0.01), and Perifovea (both *p* < 0.05) except the ParaFovea (both *p* > 0.05) was significantly reduced in the NODE and ODE groups, and the VD of the whole image, Fovea, and Perifovea was obviously decreased in the ODE group compared with the NODE group (all *p* < 0.05).

**Table 3:** The comparisons of macular retinal and choroidal microvascular density.

Variables		CRFs		Post-Hoc Analysis *p*-Values			
	Normal	NODE	ODE	*p*	Normal vs. NODE	Normal vs. ODE	NODE vs. ODE
SLR							
Whole Image	49.82 ± 3.38	42.44 ± 5.40	42.51 ± 5.37	<0.001	<0.001	<0.001	0.979
Fovea	18.92 ± 11.12	16.05 ± 4.66	15.34 ± 5.26	0.225	0.160	0.090	0.671
ParaFovea	50.33 ± 9.43	43.14 ± 8.06	43.50 ± 6.25	0.001	0.003	0.001	0.883
PeriFovea	50.70 ± 3.57	44.21 ± 5.12	44.78 ± 4.98	<0.001	<0.001	<0.001	0.748
DLR							
Whole Image	51.05 ± 6.23	45.71 ± 6.66	46.31 ± 5.48	0.001	0.004	0.003	0.772
Fovea	34.37 ± 9.36	33.51 ± 8.77	33.48 ± 8.78	0.911	0.730	0.731	0.996
ParaFovea	54.16 ± 6.83	52.08 ± 4.10	53.09 ± 4.90	0.349	0.147	0.488	0.495
PeriFovea	51.86 ± 6.96	44.59 ± 7.00	46.46 ± 5.15	<0.001	<0.001	0.001	0.355
CC							
Whole Image	70.23 ± 2.47	68.04 ± 3.73	63.09 ± 6.51	<0.001	0.029	<0.001	**0.004**
Fovea	70.17 ± 3.29	65.64 ± 6.14	58.99 ± 9.64	<0.001	0.003	<0.001	**0.010**
ParaFovea	68.21 ± 3.63	66.15 ± 4.15	66.01 ± 5.62	0.108	0.072	0.118	0.923
PeriFovea	70.88 ± 2.52	69.04 ± 3.67	66.17 ± 3.50	<0.001	0.042	<0.001	**0.007**

Data are shown as the mean ± SD. SLR, superficial layer; DLR, deep layer; CC, choriocapillaris.

### The correlation between vessel densities and visual functional parameters

The correlation of macular whole-image VD in the SLR (SLR-mwiVD), DLR (DLR-mwiVD), and CC (CC-mwiVD) with visual functional parameters is shown in **Table 4**. The BCVA, VF-PSD, and P100 latency were negatively correlated with all parameters of VD (all *p* < 0.01). The VF-MD, P100 amplitude, and HRR scores were positively associated with all parameters of VD (all *p* < 0.05)

**Table 4:** The correlation between microvascular density and visual function parameters.

Variables	SLR-mwiVD	DLR-mwiVD	CC-mwiVD
**BCVA**	**−0.536 (<0.001)**	**−0.545 (<0.001)**	**−0.398 (<0.001)**
**VF-MD**	**0.373 (0.001)**	**0.440 (<0.001)**	**0.378 (0.001)**
**VF-PSD**	**−0.425 (<0.001)**	**−0.436 (<0.001)**	**−0.534 (<0.001)**
**P100** latency	**−0.441 (<0.001)**	**−0.328 (0.004)**	**−0.337 (0.003)**
**P100** amplitude	**0.512 (<0.001)**	**0.268 (<0.020)**	**0.310 (<0.007)**
**HRR** scores	**0.571 (<0.001)**	**0.564 (<0.001)**	**0.417 (<0.001)**

Data are shown as the mean ± SD. SLR, superficial layer; DLR, deep layer; CC, choriocapillaris; mwiVD, macular whole-image vessel density.

## Discussion

Our study demonstrated that SLR-mwiVD, DLR-mwiVD, and CC-mwiVD were decreased in CRF patients with or without ODE compared with normal, and there was no difference in these parameters, except CC-mwiVD between the ODE and NODE groups. The CC-mwiVD in the ODE group was significantly reduced compared with that in the NODE group. All these VD parameters were negatively correlated with BCVA, VF-PSD, and P100 latency and positively associated with VF-MD, P100 amplitude, and HRR scores.

CRFs were first described in 1884 by Nettleship and associated with a wide variety of pathological conditions, such as tumors, central serous retinopathy, choroidal naevi, and papilloedema (26). CRFs have rarely been reported in TAO patients and are often considered as an indicator of conditions that threaten vision in TAO patients, such as DON, which is the most common vision-threatening condition of TAO (7). ODE, a specific sign of DON, was reported in 56% of eyes affected by DON, and the possible mechanism was the inflammation and edema that coexist in the orbit (23, 24). Therefore, there is a logical reason to speculate that CRFs with ODE may be more serious manifestations than CRFs alone. Although many visual functional parameters in the ODE group were indeed worse than those in the NODE group, there was no significant difference in the visual functional parameters, including BCVA, VF-MD, P100 latency, and HRR scores between two groups, with the exception of VF-PSD. Considering the effect of some systemic or ophthalmic diseases on OCTA parameters, such as hypertension and high myopia, these patients were not included in this study due to the inclusion and exclusion criteria, although these diseases may have a low impact on vision. Therefore, a possible explanation is that not all patients could be included due to the inclusion and exclusion criteria in this study.

OCTA, a novel noninvasive technique, was recently adopted to quantitatively measure retinal and choroidal microvasculature in different ocular and systemic diseases, such as glaucoma (27, 28), age-related macular degeneration (29–31), optic neuropathies (32–35), leukemia (36), diabetic retinopathy (37–40), and TAO (14, 19, 20, 41, 42), and to evaluate the severity and prognosis of these diseases. Our study showed that the macular retinal VD was significantly decreased in the ODE and NODE groups compared with the normal group. Moreover, macular retinal VD was correlated with visual dysfunction in this study. Similar results have been reported in DON patients in previous studies (18, 20, 21). Not all CRF patients have DON symptoms, which are present in 47%–50% of eyes with CRFs (7) and ODE is a specific diagnostic marker of DON and could be used to diagnose as DON in this study. Our study showed no difference in the VD of the macular retina between the ODE and NODE groups. Therefore, these studies may suggest that decreased macular retinal VD in CRF patients may be valid evidence to diagnose vision-threatening TAO.

A recent study showed that choroidal RPC was significantly reduced in DON, which is correlated with VF defects (18). However, to the best of our knowledge, no study on macular choroidal changes has been reported in TAO patients with CRFs. In this study, the VD of the macular CC was significantly reduced in CRF patients with or without ODE and correlated with visual dysfunction. Therefore, decreased choroidal VD in CRF patients may be valid evidence to diagnose vision-threatening TAO. In addition, our study showed that compared with the NODE group, the macular choroidal VD was significantly reduced in the ODE group, while the retinal VD was not different. It is powerful evidence to suggest that CRFs with ODE may be more serious than CRFs alone.

The current study has some limitations. First, this was a cross-sectional study, which makes it difficult to evaluate dynamic changes in VD over the course of the disease and understand its role in the development of disease. Second, the macular choroidal VD with OCTA is determined by projection artifacts, which may interfere with the results of the study. Third, the sample size of CRF-affected eyes was also small because CRFs were rare in TAO patients, and more samples would make the results stronger.

## Conclusion

In summary, our results demonstrated a notable decrease in choroidal and retinal macular VD in patients with CRFs with or without ODE, which is correlated with visual dysfunction. The macular choroidal VD in CRF patients with ODE was significantly reduced compared with that in NODE patients and may be an alternative index to diagnose **v**ision-threatening TAO.

## Data availability statement

The original contributions presented in the study are included in the article/supplementary material. Further inquiries can be directed to the corresponding authors.

## Ethics statement

The protocol of this study was approved by the Sun Yat-sen University Sun Yat-sen Memorial Hospital Ethical Committee in the People’s Republic of China. The patients/participants provided their written informed consent to participate in this study.

## Author contributions

PZ, J-QL and Y-YP have contributed equally to the work. All authors contributed to the article and approved the submitted version.
